# The Roles of Vitamin A in the Regulation of Carbohydrate, Lipid, and Protein Metabolism

**DOI:** 10.3390/jcm3020453

**Published:** 2014-05-07

**Authors:** Wei Chen, Guoxun Chen

**Affiliations:** Department of Nutrition, University of Tennessee at Knoxville, Knoxville, TN 37996, USA; E-Mail: wchen16@utk.edu

**Keywords:** vitamin A, glucose metabolism, lipid metabolism, protein metabolism, metabolic diseases

## Abstract

Currently, two-thirds of American adults are overweight or obese. This high prevalence of overweight/obesity negatively affects the health of the population, as obese individuals tend to develop several chronic diseases, such as type 2 diabetes and cardiovascular diseases. Due to obesity’s impact on health, medical costs, and longevity, the rise in the number of obese people has become a public health concern. Both genetic and environmental/dietary factors play a role in the development of metabolic diseases. Intuitively, it seems to be obvious to link over-nutrition to the development of obesity and other metabolic diseases. However, the underlying mechanisms are still unclear. Dietary nutrients not only provide energy derived from macronutrients, but also factors such as micronutrients with regulatory roles. How micronutrients, such as vitamin A (VA; retinol), regulate macronutrient homeostasis is still an ongoing research topic. As an essential micronutrient, VA plays a key role in the general health of an individual. This review summarizes recent research progress regarding VA’s role in carbohydrate, lipid, and protein metabolism. Due to the large amount of information regarding VA functions, this review focusses on metabolism in metabolic active organs and tissues. Additionally, some perspectives for future studies will be provided.

## 1. Introduction

The prevalence of overweight and obesity has become a public health concern around the world [1]. According to the World Health Organization’s 2010 report, 35% of adults aged 20 years and older worldwide were clinically overweight (body mass index, BMI ≥ 25 kg/m^2^) based on data from 2008. More than half a billion adults in the same category were obese (BMI ≥ 30 kg/m^2^) [2]. Moreover, 2.8 million deaths are linked to raised BMI each year [2]. Overweight and obesity are associated with an increased risk of developing an assortment of metabolic diseases, which mainly include hypertension, type 2 diabetes mellitus, non-alcoholic fatty liver disease, coronary heart disease, and stroke [1,3]. The implications of all those health conditions have brought gloomy biomedical and socioeconomic consequences to the well-being of the global population [3].

Both genetic and nutritional/environmental factors are instrumental to the development of obesity and its related metabolic diseases [4,5]. Mutations in the appetite-regulating hormones (e.g., leptin and proopiomelanocortin) and their corresponding receptors (e.g., leptin receptor and melanocortin 4 receptor, respectively) result in early onset of obesity and obesity-associated metabolic dysregulation in both humans and experimental rodent models [6]. Given the increasing numbers of the monogenic contributors, the majority of the obesity cases in the human population are believed to be polygenic [6]. On the other hand, overnutrition related to unhealthy eating habits probably drives the rising global prevalence of overweight and obesity [7,8]. In spite of the self-evident link between overnutrition and obesity, the underlying mechanisms that cause obesity and its related metabolic diseases due to excessive intake of nutrients have not been fully understood.

The utilization of dietary macronutrients requires coordinative regulation of the metabolic pathways in different organs and tissues of the body. In a fed state, excessive dietary macronutrients are conserved in the form of glycogen and triglycerides (TG) for future energy needs. During fasting, glycogen and TG are, respectively, broken down into glucose and fatty acids (FAs) to meet the energy needs of different organs and tissues in the body. These processes are coordinately regulated by both hormonal and nutritional stimuli in response to feeding and energy states so that the body reaches energy homeostasis [4,5]. Dysregulated hormonal balance and nutrient metabolism not only disrupt the energy homeostasis, but also predispose individuals to developing obesity and related metabolic diseases [5].

Insulin, secreted from pancreatic β-cells in response to physiological stimuli, is an important peptide hormone in anabolism [9]. Upon secretion, it binds to the insulin receptor on cell membranes and exerts its action through a signal transduction cascade that is mainly composed of kinases and phosphatases. The binding causes conformational changes of the insulin receptor and transphosphorylation of the β-subunit tyrosine kinase [10,11], which initiates the signal transduction cascade [12]. The signal is transduced by multiple components in a complex network containing multiple kinases and phosphatases [13,14]. The activation of the insulin signaling pathway results in changes in the activities and/or the expression levels of enzymes involved in nutrient metabolism. These subsequently alter the metabolic state of tissues and organs in the body. For example, insulin promotes glycolysis, glycogenesis, and lipogenesis, and, at the same time, suppresses gluconeogenesis in the liver [14]. However, in overweight and obese individuals, a certain dose of insulin only produces subnormal physiological responses. This observation is regarded as insulin resistance, which occurs concurrently with profound alterations in glucose and lipid metabolism [9,15,16]. Even though the molecular mechanisms leading to insulin resistance have not been fully understood, it is postulated that systemic inflammation, dysregulated lipid metabolism, and gastrointestinal dysbiosis, triggered by overnutrition, interplay with each other and result in the impairment of insulin action [16].

Overnutrition not only provides excessive energy from macronutrients, but also superfluous amounts of vitamins and essential factors, which have regulatory roles. How micronutrients, such as vitamin A (VA; retinol), regulate the homeostasis of macronutrients is still an ongoing research topic. As an essential and lipophilic micronutrient, VA plays a key role in the general health of an individual [5]. This review summarizes recent research progresses regarding the role of VA in carbohydrate, lipid and protein metabolism in the liver, pancreas, skeletal muscle, and adipose tissues, which are major active players in glucose and FA metabolism.

## 2. Overview of VA and Its Metabolism

VA is important for a myriad of physiological functions, including vision formation, immune response, cell differentiation and proliferation, embryonic development and metabolism [17]. The investigation of its pleiotropic effect on animals resulted in the discovery of the retinoid signaling pathway by which VA exerts its function by modulating gene expression in target cells [18,19]. Dietary molecules with VA activities exist in two forms: provitamin A (carotenoids and cryptoxanthins) and preformed VA (retinol or retinyl esters). Provitamin A molecules are mainly found in plants. Herbivorous and omnivorous animals convert provitamin A molecules into retinol before storing it as retinyl esters in different organs and tissues, depending on the species [20]. Retinol and retinyl esters are then introduced into the food chain and transferred to carnivorous and omnivorous animals at the higher tiers of the food chain [21].

**Figure 1:**
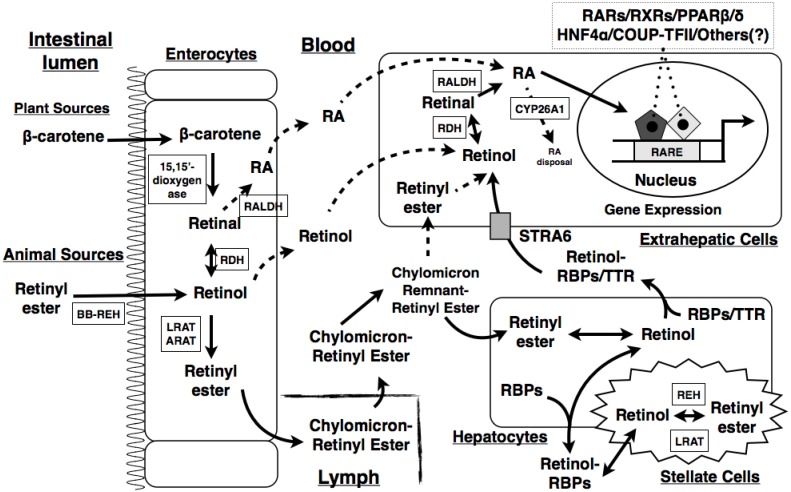
Schematic representation of vitamin A (VA) digestion, absorption, transport, and metabolism in the body. Dietary VA from plant and animal sources is digested in the intestinal lumen and absorbed by enterocytes via different mechanisms. Within the enterocytes, the dietary VA is converted into retinyl esters, which are packaged into chylomicrons for the secretion into the lymph and eventually enter the circulation. The lipoprotein lipase hydrolyzes triglycerides (TGs) on the chylomicrons to produce chylomicron remnants. The retinyl ester-containing chylomicron remnants are eventually taken up by hepatocytes, where the retinyl esters are again hydrolyzed into retinol. The released retinol can be transported to target cells and catabolized into retinal, retinoic acid (RA), or other metabolites. Excessive retinol is re-esterified into retinyl esters, which are stored in stellate cells. BB-REH, brush-border retinyl ester hydrolase; LRAT, lecithin:retinol acyltransferase; ARAT, acyl-CoA:retinol acyltransferase; RBP, retinol binding protein; RDH, retinol dehydrogenase; RALDH, retinal dehydrogenase; REH, retinol ester hydrolase; STRA6, stimulated by retinoic acid 6; RA, retinoic acid; RARE, retinoic acid responsive element; RAR, retinoic acid receptor; RXR, retinoid X receptor; TTR, transthyretin; PPAR, peroxisome proliferator-activated receptor; HNF4α, hepatocyte nuclear factor 4α; COUP-TFII, chicken ovalbumin up-stream transcription factor II.

After consumption (Figure 1), dietary retinol, retinyl esters, and carotenoids are released from the digested food matrix in the gastrointestinal tract. In the intestinal lumen, the brush-border retinyl ester hydrolase hydrolyzes retinyl esters into retinol and free FAs [22,23]. Due to their lipophilic characteristic, these molecules are incorporated into micelles in the intestinal lumen prior to absorption. Carotenoids enter enterocyte (intestinal mucosal cell) via passive diffusion, which neither requires cell membrane transporters nor energy [24,25]. Retinol is thought to be taken up into enterocytes by a brush border membrane transporter via facilitated diffusion [26].

Inside the enterocytes, the majority of β-carotene (major dietary carotenoid) will be symmetrically cleaved by 15,15′-dioxygenase to generate retinal [27]. Residual intact β-carotene can be delivered to the liver, where the same enzyme in hepatocytes catalyzes the cleavage reaction [25]. Aside from the symmetrical cleavage, β-carotene can also be asymmetrically cleaved at the 9′,10′ double bond by a specific enzyme in the enterocyte [28,29]. The resulting retinal from the cleavage of the β-carotene is readily reduced to retinol, which converges into the retinol repertoire from the hydrolysis of retinyl esters [30]. The majority of the retinol in the enterocyte is re-esterified into retinyl ester by lecithin:retinol acyltransferase [31] (LRAT) or acyl-CoA:retinol acyltransferase [32] (ARAT), and then incorporated into chylomicrons for delivery [30].

In the circulation, retinyl ester-containing chylomicrons interact with the lipoprotein lipase at the inner lining of endothelial cells of the vascular system, which gradually hydrolyzes the TGs on the chylomicrons to free FAs. This process decreases the size of the chylomicrons and converts them into chylomicron remnants. The retinyl ester-containing chylomicron remnants are eventually taken up by hepatocytes, where retinyl esters are again hydrolyzed into retinol. The released retinol can be transported to target cells and catabolized into retinal, retinoic acid (RA), or other metabolites for the different physiological functions. Excessive retinol is re-esterified into retinyl esters, which are stored in stellate cells inside the liver [5]. A combination of retinol and RA is shown to increase retinol uptake and esterification in the lung [33]. A portion of retinyl esters is absorbed by extrahepatic tissues [34].

Retinol derived from the liver is associated with retinol binding protein 4 (RBP4) and transthyretin (TTR) as a complex for the delivery to extrahepatic tissues. The uptake of retinol in the holo-RBP4-TTR complex is supposedly mediated by a receptor on the cell’s membrane. Stimulated by retinoic acid gene 6 (STRA6), a membrane receptor protein, is identified from bovine retinal pigment epithelium cells due to its high affinity to bind to RBPs [35]. Mutations in the human *STRA6* gene cause Matthew-Wood Syndrome [36,37]. However, the deletion of the *Stra6* gene in mice does not cause any apparent developmental or growth defect [38,39,40]. Interestingly, other than retinal pigment endothelium abnormalities [38,39,40], the levels of retinol and retinyl esters in the plasma and other tissues in *Stra6*^−/−^ mice are similar to those in wild type control mice [38]. *Stra6*^−/−^ mice have normal glucose tolerance [38,39]. When *Stra6*^−/−^ mice were fed a VA deficient diet for 5 weeks, they were shown to have less retinol and retinyl ester levels in the white adipose tissue, kidney, heart, and testis than wild type control mice, indicating that STRA6 is responsible for part of the retinol uptake in these tissues [38]. The deletion of *Stra6* in mice reduces insulin resistance mediated by the injection of holo-RBP and feeding of a high-fat diet, suggesting that STRA6 functions as a receptor to mediate RBP-induced insulin resistance [38]. These results indicate that STRA6 is not the only pathway for retinol transport, and other transporters or mechanisms may exist for retinol uptake in cells.

Many of VA’s physiological functions are mediated by RA. The generation and disposal of RA are highly regulated processes involving complex retinoid binding proteins and metabolic enzymes [41,42]. In essence, retinol can be reversibly oxidized into retinal by retinol dehydrogenases (RDHs), and further irreversibly oxidized into RA by retinal dehydrogenases (RALDHs), as reviewed in [41]. *In vitro* biochemical experiments have so far identified almost a dozen enzymes with retinol oxidation capabilities, but only RDH1, RDH10, and dehydrogenase/reductase member 9 (DHRS9) are verified and supported by *in vivo* evidence [41]. Comparatively, four RALDHs have been identified, which are expressed and regulated in a tissue specific manner [41]. RALDH1 is universally expressed and accounts for over 90% of retinal oxidation capability in rat liver and kidney [43]. Despite its ubiquitous expression profile, RALDH1 knockout mice do not manifest developmental differences compared to their wild type counterparts [44]. Interestingly, RALDH1 knockout mice are resistant against high-fat diet induced glucose intolerance and obesity [45,46]. In contrast to RALDH1, RALDH2 knockout mice are embryonically lethal. This suggests that RALDH2 is responsible for the local production of RA during embryo development [47]. RALDH3 does not recognize 9-*cis* retinal as a substrate, whereas RALDH4 only recognizes 9-*cis* retinal as a substrate [48,49]. The selective recognition of substrates suggests distinctly different functions of the two enzymes *in vivo*. However, more research is necessary to elucidate the underlying mechanisms [48,49]. The catabolic cytochrome P450 (CYP) family of enzymes partially contributes to RA homeostasis. These enzymes require NADPH and dioxygen to convert RA into a myriad of end products, including 4-hydroxy-RA, 4-oxo-RA, and 18-hydroxy-RA [50]. A representative member of the family, CYP26A1, was cloned and investigated for its role in the regulation of RA homeostasis in animals [51,52,53]. CYP26A1 levels are low in VA deficient animals, but CYP26A1 is substantially induced by RA or a VA sufficient diet [52]. CYP26A1 null mice are embryonically lethal, showing phenotypes similar to wild type mice treated with excessive RA [54]. These data implicate an autoregulatory mechanism by which CYP26A1 controls RA catabolism to maintain RA levels *in vivo*.

After RA molecule synthesis, they translocate to the nucleus, where they bind to and activate two subfamilies of nuclear receptors known as the retinoid acid receptors (RARs) and retinoid X receptors (RXRs). Nuclear receptors are ligand-activated transcription factors involved in a variety of physiological processes [55]. RARs and RXRs each contain three isoforms that have different tissue and developmental expression profiles [55]. It has been thought that all-*trans* RA is an exclusive ligand for RARs, whereas 9-*cis* RA is a common ligand for both RARs and RXRs [56]. Transcription factors mediating RA activity can recognize short consensus sequences, which are termed retinoic acid response elements (RAREs), located in the promoter region of the RA responsive genes. Depending on the specific gene, RXR can bind to RARE as a homodimer or a heterodimer with RAR or other nuclear receptors [19]. As shown in Figure 1, in addition to RARs and RXRs, RA signals have been suggested to be mediated by other nuclear receptors, such as peroxisome proliferator-activated receptor (PPAR) β/δ, hepatocyte nuclear factor 4α, and chicken ovalbumin up-stream transcription factor II [5,57]. The binding of their specific ligands to the nuclear receptors will help recruit corresponding transcriptional coactivators and dissociate transcriptional corepressors in the general transcription machinery, leading to the regulation of RA-targeted gene expression.

## 3. VA and Growth, Appetite, Taste and Olfaction

VA was first recognized as an essential factor for animal growth. Following the withdrawal of VA from the diet, growth retardation is the earliest and most reproducible sign of deficiency in many experimental animals [58]. Rats fed on a VA deficient diet upon weaning (about three weeks old), manifest cessation of growth and significant weight loss later on [59,60]. The reduction of body mass can be prevented by RA supplementation. However, once RA is removed from those rats fed on the VA deficient diet, the weight loss will reappear in a couple of days [61,62]. These results demonstrate that VA is important for normal growth and development of animals.

In comparison to rats, mice are more resistant to the development of VA deficiency. A normal growth curve is usually observed in weaning mice with sufficient hepatic VA storage, despite being sustained on a VA deficient diet [63,64]. In one study, a significant weight loss was achieved in mice fed on a VA deficient diet only when the VA storage was pre-depleted prior to weaning [63]. This suggests that mice might have special mechanisms to maintain VA homeostasis or they might use VA more efficiently. As a result, experimental conclusions obtained in mice cannot be directly extrapolated to rats or humans in terms of VA and its effect on growth and metabolism.

VA deficient animals exhibit decreased appetite and food intake during the period of growth cessation [59,60]. The underlying molecular mechanisms by which VA regulates energy intake remain elusive. Since leptin, an adipocyte-derived peptide hormone, exerts its action in the brain to regulate appetite, energy expenditure, and metabolism [65], some researchers investigated the effects of VA on the expression of leptin in rodents. The results showed that both RA treatment and VA supplementation were able to decrease leptin mRNA in adipocytes, which, in turn, decreased serum leptin levels [66,67]. Interestingly, the energy intake of these rodents did not change during the course of VA supplementation [68]. These data suggest that the VA-induced decrease in leptin levels does not correlate with food intake in these animals [68].

VA has also been implicated in the normal functions of the taste and olfactory system. VA depletion causes the loss of both preference to sodium chloride and aversion to quinine in rats, and these abnormal responses could be restored by VA supplementation [69]. One study also shows that the response to sweetness in VA deficient rats is impaired [70]. Furthermore, VA supplementation can improve the impairment of taste and olfaction in patients with cirrhosis [71]. Despite these many observations, the mechanism is not clear. Keratin infiltration into the taste buds is proposed to be a mechanism by which VA deficiency affects taste in rats [69]. But the physical change could not be confirmed in all VA deficient rodent models [70].

## 4. VA and Plasma Parameters: Clinical Evidence

The association between VA and metabolism was discovered from clinical observations in human subjects with type 2 diabetes. Biopsies of the liver of diabetic patients showed that the hepatic VA content was twice as much compared with healthy individuals [72]. Independent studies carried out more than eighty years ago also found that about 85% of adult patients with type 2 diabetes had elevated plasma carotene levels, and more than 10% were clinically diagnosed with xanthosis [73,74]. Similar results were obtained to a lesser degree in type 2 diabetic children, but their plasma VA levels were found to be subnormal [75]. Additionally, reduction of serum retinol and RBP levels was found in type 1 diabetic patients [76]. These data collectively suggest that VA plays important roles in metabolic homeostasis.

Recently, RBP4, a serum retinol transporter, was shown to be elevated in insulin resistant and type 2 diabetic subjects [77,78,79]. Multiple single nucleotide polymorphisms (SNP) of RBP4 were also discovered in the human genome, which predict the susceptibility to insulin resistance, obesity, and type 2 diabetes [80,81]. Despite suggesting a positive association, several clinical studies did not identify significant associations between changes in RBP4 levels and metabolic diseases [82,83,84]. It is worth to note that elevation of plasma RBP4 levels reduced insulin sensitivity in mice, and RBP4 knockout mice had improved insulin sensitivity [85]. When fenretinide, a synthetic retinoid derivative, was used to disrupt the association of RBP4 and TTR, insulin sensitivity in diet-induced obese mice was improved [85]. However, when a specific compound was developed to disrupt the RBP4 and TTR interaction and successfully reduced the plasma RBP4 level, insulin sensitivity in the insulin resistant mice was not improved, suggesting that another mechanism may be responsible for the fenretinide-mediated improvement of insulin sensitivity in mice [86]. Moreover, the improvement of insulin sensitivity in RBP4 knockout mice could not be observed in this study [86]. Further studies are needed to understand the role of VA in insulin resistance.

The association between VA and plasma lipid metabolism was also observed in patients taking retinoid drugs. Medical administration of isotretinoin (13-*cis* RA) results in hypertriglyceridemia in human subjects with acne [87]. Treatment of patients with acute promyelocytic leukemia with all-*trans* RA leads to weight gain and elevation of plasma TG and cholesterol levels [88,89]. It is postulated that the dysregulated plasma lipid levels are caused by the RA-induced apolipoprotein CIII expression [90]. Apolipoprotein CIII is regarded as an inhibitor of lipoprotein lipase activity [91]. On the contrary, long-term administration of fenretinide, a synthetic retinoid drug currently in phase II clinical trial evaluation, could prevent diet-induced obesity, insulin resistance, and hepatosteatosis [92,93]. As more and more retinoid drugs are synthesized and tested, it would be interesting to see how they affect human metabolism in any given health condition.

## 5. VA and Metabolism in the Liver

### 5.1. VA and Hepatic Carbohydrate Metabolism

The liver plays a leading role in the regulation of carbohydrate metabolism in response to the feeding and fasting statuses. Experiments in rodents show that both hypervitaminosis A and hypovitaminosis A affect carbohydrate metabolism in the liver [4].

Glycogen is a crucial polysaccharide in which intracellular glucose is stored. Hepatic glycogen synthesis and degradation contributes to plasma glucose homeostasis. In VA deficient rats, the liver glycogen content is abolished due to decreased glycogenesis from acetate, lactate, and glycerol, rather than directly from glucose [94,95]. The decreased glycogenesis can be recovered by the administration of glucocorticoid hormones [95]. Since VA deficient animals have lower adrenal steroid production, it is possible that VA deficiency partially affects glycogen metabolism via decreasing glucocorticoid hormone synthesis [95]. In hypervitaminotic A rats, the liver glycogen deposition in the fed state is almost the same or slightly lower than in rats fed on a chow diet [96,97]. However, the hepatic glycogenesis of hypervitaminotic A rats after fasted for 18 to 20 h is significantly higher than in rats fed chow diet [97]. This suggests that excessive VA intake for a short-term can prevent the hepatic glycogenolysis under the fasting condition. Additionally, excessive VA can also enhance the hepatic glycogenesis from glucose after refeeding in normal rats, but not in adrenalectomized rats [98]. This suggests that adrenal hormones are involved in the VA-regulated hepatic glycogen metabolism [98].

Glycolysis is a series of enzymatic reactions, which break down glucose to produce pyruvate and generate ATP and NADH. This important carbohydrate metabolic pathway is influenced by VA status. In VA deficient hamsters, the hepatic generation of glucose-6-phosphate was decreased by up to 90% due to reduced glucokinase (gene *Gck*) activity [99]. Similarly, the generation of mannose-6-phosphate was also decreased due to impaired hexokinase activity [99]. On the contrary, hypervitaminosis A does not affect the enzymatic activities of hepatic glucokinase or hexokinase [100]. Instead, excessive VA can decrease the enzymatic activities of phosphofructokinase and pyruvate kinase [100]. Additionally, *Gck* mRNA levels in the liver of VA deficient rats are lower than in VA sufficient counterparts [60]. Retinoids not only increase hepatic *Gck* expression in rats, but also synergize with insulin to induce *Gck* expression in primary rat hepatocytes [101]. These observations suggest that VA is an important regulator of hepatic glycolytic enzymes.

Gluconeogenesis is a series of enzymatic reactions, which utilize non-carbohydrate substrates (e.g., lactate, pyruvate, amino acids) to generate glucose. This metabolic pathway is very important in maintaining fasting blood glucose levels in animals. Experiments with VA deficient rats show that the enzymatic activities of glucose-6-phosphatase and fructose-1,6-bisphosphatase were decreased in the liver, showing an inhibition of hepatic gluconeogenesis [102]. Comparably, hypervitaminotic A rats have elevated hepatic enzymatic activities of the cytosolic form of phosphoenolpyruvate carboxykinase (PEPCK, gene *Pck1*), glucose-6-phosphatase, and fructose-1,6-bisphosphatase [103,104]. In addition, the VA-mediated increase in gluconeogenesis is abolished in adrenalectomized rats [105], showing the requirement of adrenal hormones in the process. The regulation of gluconeogenesis by VA can also occur at the transcription level of *Pck1* expression in the liver. RA not only induces *Pck1* expression in primary rat hepatocytes, but also attenuates insulin-suppressed *Pck1* expression [106]. In fact, insulin can inhibit RA-activated RXRs, but not RARs in the *Pck1* promoter. This mechanism underlies the production of *Pck1* mRNAs in the presence of RA, despite the insulin-suppressed state [106]. Additionally, a *Pck1* transgenic mice model shows that all-*trans* and 9-*cis* RAs differentially regulate hepatic *Pck1* expression in the periportal region of the liver acinus [107]. This retinoid-regulated *Pck1* expression is purportedly mediated by different RAREs and nuclear receptors at its promoter [107,108].

### 5.2. VA and Hepatic Protein Metabolism

Ever since the increased nitrogenous metabolism was first described in VA deficient rats [109], many animal models were used to investigate the effects of VA on protein metabolism. In young male rats fed on a VA deficient diet, urinary nitrogen excretion is increased with a concurrent negative nitrogen balance [110]. It is in line with the observation of increased plasma urea in VA deficient adult rats [111]. Interestingly, these physiological changes cannot be obtained in VA deficient female rats, indicating a sex difference in the effects of VA on protein metabolism [112]. Additionally, VA deficiency increases both the mRNA levels and enzyme activities of most urea cycle enzymes in the rat liver [111]. These data suggest that VA deficiency increases protein catabolism by upregulating the enzymes of the urea cycle in both the liver and kidney.

The effect of VA deficiency on protein anabolism is controversial. The analysis of the incorporation rate of ^14^C-leucine into proteins in both VA deficient and chow-fed rats shows that VA deficiency does not adversely affect protein synthesis [113]. However, *in vitro* protein synthesis assay using isolated rat liver ribosomes show the opposite effect. Cell precipitates from the pH 5.1 fraction of the VA deficient rat liver homogenate exhibit enhanced protein synthesis capability [114]. The reason for these conflicting results is not known.

Very few studies looked at the effects of hypervitaminosis A on protein metabolism in animals. It is proposed in one study that 400 times the normal VA dose causes toxicity-induced weight loss and negative nitrogen balance [115]. However, the molecular mechanism by which the VA toxicity offsets the nitrogen balance *in vivo* is unknown.

### 5.3. VA and Hepatic Lipid Metabolism

Dysregulation in the metabolism of either VA or lipids could negatively affect the metabolism of the other. On the one hand, long-term retinoid drug users and people who take excessive amounts of VA supplements exhibit symptoms of hypercholesterolemia, hypertriglyceridemia, and high serum low density lipoprotein levels [116,117,118]. On the other hand, patients with severe type V hyperlipoproteinemia-associated hypertriglyceridemia have an increased risk for developing hypervitaminosis A [119]. The mechanisms underlying these phenomena have been actively investigated in rodent models. Oral administration of large doses of retinol leads to the accumulation of lipid droplets in the rat liver [120]. Overdose of retinol or retinyl palmitate also causes the elevation of hepatic cholesterol, FA, and TG contents in different strains of rats [121,122,123].

It has been shown that hypervitaminotic A rats have increased rates of hepatic FA oxidation and ketogenesis [123]. On the other hand, the hepatic TG synthesis rate is greatly enhanced in hypervitaminotic A rats. This is due to the increased incorporation rate of palmitate into TGs and the formation of glycerophosphate from glucose [122,123]. Interestingly, the rate of hepatic TG secretion is not changed in hypervitaminotic A rats [124]. These data suggest that VA increases hepatic lipid synthesis to an extent much greater than that it elevates hepatic lipid oxidation. The net result is the accumulation of lipids in the liver. A further investigation shows that the hyperlipidemic effect of excessive intake of VA cannot be observed in adrenalectomized rats, showing the involvement of adrenal hormones [122].

Contrary to the hyperlipidemic effect of hypervitaminosis A, VA deficiency causes a partial hypolipidemic effect in rodents. In general, VA deficient rats have decreased hepatic phospholipid content and decreased serum levels of TG, cholesterol, and high density lipoprotein [60,125]. Interestingly, these rats manifest unaltered hepatic contents of TG and cholesterol [123,126,127,128]. Depending on the severity of VA deficiency in the animals, not all of the above-mentioned symptoms can be observed. The partial hypolipidemic effect of VA deficiency in rodents may be caused by a decreased FA synthesis activity in the liver and impaired cholesterol synthesis from mevalonate [125,128]. However, it is worth mentioning that the VA deficiency-induced body mass loss and food intake drop may also contribute to the hypolipidemic symptoms. Recent evidence shows that the decrease in total percentage of body fat is similar between VA deficient rats and their counterparts pair-fed on a VA sufficient diet [129]. In drastic contrast to VA deficient rats, VA deficient mice show TG accumulation in the liver, resulting from reduced expression levels of mitochondrial FA oxidation genes [130]. The altered expression of these genes in the liver may be attributed to the downregulation of the hepatic *PPARα* gene [130]. Accordingly, RA treatments in mice increase hepatic FA oxidation, which has been reviewed in detail elsewhere [131]. Available data suggest that the dyslipidemia in VA deficient and hypervitaminotic A animals is a multicausal effect. The distinct responses of hepatic lipid metabolism to VA status in mice and rats deserve further investigation.

Hepatic FA synthesis is governed by an allotment of lipogenic genes, including acetyl-CoA carboxylase (ACC, gene *Acc*) and FA synthase (FAS, gene *Fas*). The transcription of hepatic *Acc* and *Fas* is controlled by a transcriptional factor termed sterol-responsive element binding protein 1c (SREBP-1c, gene *Srebp-1c*) [132]. In primary rat hepatocytes, insulin induces *Srebp-1c* expression through transcription factors associated with the liver X receptor elements in the *Srebp-1c* gene promoter [133]. Interestingly, the corresponding liver X receptor elements in the *Srebp-1c* promoter are also identified as RAREs [134]. This finding provides a possible mechanism by which RA synergizes with insulin to induce hepatic *Srebp-1c* expression [134]. It also showcases how nutritional and hormonal factors converge at the transcriptional level to regulate *de novo* FA synthesis in the liver.

### 5.4. VA and Mitochondrial Functions in Hepatocytes

Mitochondria are the powerhouses of the cell in which the tricarboxylic acid cycle (TCA cycle) and coupled-oxidative phosphorylation conjointly generate ATP for physiological events. The TCA cycle consists of a series of enzymatic reactions that generate reducing agents (NADH and FADH2) by utilizing acetyl-CoA derived from monosaccharides, amino acids, and FAs. The reducing agents ultimately provide electrons, which are consumed in the coupled oxidative phosphorylation process to reduce O_2_ and generate ATP.

In the rodent liver, both hypo- and hypervitaminosis A are implicated in the uncoupled oxidative phosphorylation in mitochondria [135,136,137]. On the one hand, the dioxygen consumption by liver homogenates of hypo- and hypervitaminotic A rats is significantly increased upon the supply of some TCA intermediates [135,136]. On the other hand, the capacity for oxidative phosphorylation is severely impaired in liver mitochondria of hypo- and hypervitaminotic A rats without affecting ATPase activity [137]. These data suggest that deficient or excessive VA status may increase basal energy metabolism in the liver.

## 6. VA and Islets of Langerhans in the Pancreas

Islets of Langerhans, which account for less than 2% of the total pancreas mass, produce and secrete peptide hormones from five different types of specialized cells, including α-, β-, polypeptide (PP-), δ-, and ε-cells [138,139]. Among all the hormones produced by islets, glucagon from α-cells and insulin from β-cells are of great clinical interest due to their concerted roles in the regulation of blood glucose levels. Glucagon promotes hepatic glucose production, glycogenolysis, and ketone production in response to hypoglycemia, whereas insulin promotes glucose disposal, glycogenesis, and lipogenesis in response to the increase of plasma glucose levels [140].

The secretion of glucagon is controlled by the autonomous nervous system, direct action of glucose on α-cells, and indirect effects of paracrine factors from non-α-cells on α-cells [141]. On the other hand, the secretion of insulin is mainly stimulated by the influx of glucose into β-cells. Glucose metabolism in β-cells leads to elevated ATP/ADP ratios, which in turn, inhibit the ATP-sensitive potassium channels on the cell membrane. The inhibition depolarizes the plasma membrane, which results in the secretion of insulin. In addition to glucose, amino acids and neural stimuli can also stimulate insulin secretion [142].

It has been shown that VA and its metabolites can affect the secretion of glucagon. In VA deficient rats, impaired glucagon secretion occurs from early stages of deficiency, and the impairment cannot be rescued by RA replenishment [143]. This demonstrates a critical physiologic role of VA in the normal function of α-cells. Interestingly, in the isolated intact rat islets and glucagon secreting cell lines, both retinol and RA inhibit glucagon secretion in a dose-dependent manner [144]. This result suggests that the acute effects of VA on glucagon secretion *in vitro* do not correlate with its physiological effects *in vivo*.

VA deficiency has been shown to decrease β-cell functions in rats in two ways. First, it reduces the β-cell mass in fetal islets by reducing fetal β-cell replication [145]. Second, it impairs glucose-stimulated insulin secretion (GSIS) from β-cells [146]. In comparison, the effects of VA and its metabolites on isolated islets and insulin secreting cells are both chemical- and dosage-dependent. For example, retinol at 10^−7^ M stimulates GSIS from isolated rat islets, which is in opposition to the inhibitory effect of retinol at 10^−4^‒10^−5^ M [147]. Additionally, all-*trans* RA potentiates GSIS [148,149,150], whereas 9-*cis* RA inhibits GSIS [151] from rodent islets and INS-1 cells. Cellular retinol-binding protein I knockout mice exhibit increased levels of 9-*cis* RA in the pancreas and reduced GSIS [152]. It is also observed that all-*trans* RA increases *Gck* mRNA levels and induces enzyme activity of glucokinase [150], whereas 9-*cis* RA treatment is associated with decreased GLUT2 and glucokinase activities in rodent islets [151]. Given the fact that retinoid receptors require preferential ligands to exert their full function, these results suggest that the production and balance of VA and its metabolites are critical for the normal functioning of β-cells.

## 7. VA and Metabolism in Adipose Tissues

### 7.1. VA and Metabolism in White Adipose Tissue

White adipose tissue not only stores excessive energy as TGs, but also secretes important adipocyte-derived hormones and cytokines to regulate whole body energy metabolism. VA and its metabolites have determinant effects on the metabolic homeostasis in white adipose tissue.

First, VA status has been shown to affect adiposity in animals. In VA deficient rats, the loss of body fat deposit mass is reflected in the decreased total body mass [110]. Since VA deficient rats have significantly lower body mass compared to their VA sufficient diet pair-fed counterparts, it suggests that the reduction of fat mass in VA deficient rats cannot be fully attributed to the VA deficiency associated with the reduction of food intake [110]. In Zucker lean rats, VA deficiency decreases the epididymal fat mass and its fat mass/body mass ratio [60]. In Zucker fatty rats, a genetic rat model of obesity, VA deficiency retards the development of obesity [60]. Additionally, hypervitaminotic A rats have decreased release of FAs from white adipocytes, whereas VA deficient rats exhibit increased rates of lipolysis in white adipose tissue [153]. Despite the fact that these findings support VA’s role in maintaining adiposity, other studies have reported different observations. Mice on a VA deficient diet for 10 weeks show increased adiposity [154]. More importantly, in humans, low plasma VA status has been associated with overweight and obesity [155,156]. These divergent data warrant further research into the relationship between VA status and adiposity in animals.

**Figure 2:**
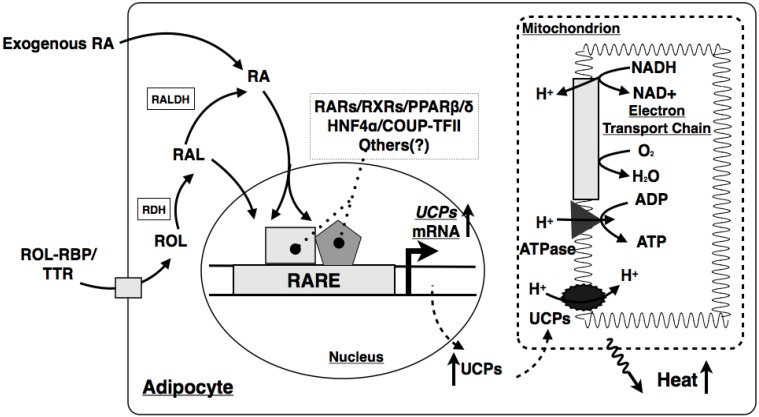
Mechanism by which RA induces thermogenesis in adipocytes. Stepwise enzymatic reactions convert retinol into retinal, and then retinal into RA in the adipocytes. RA enters the nucleus, where it modulates the expression of UCP genes by activating the corresponding nuclear receptors in the promoters of the genes. Increased UCP proteins uncouple oxidative phosphorylation from ATP production. The reduced H^+^ gradient across the mitochondrial membranes leads to the increase of thermogenesis in the adipocytes. ROL, retinol; RBP, retinol binding protein; TTR, transthyretin; RAL, retinal; RDH, retinol dehydrogenase; RALDH, retinal dehydrogenase; RA, retinoic acid; RARE, retinoic acid responsive element; RAR, retinoic acid receptor; RXR, retinoid X receptor; UCP, uncoupling protein; PPAR, peroxisome proliferator activated receptor; HNF4α, hepatocyte nuclear factor 4α; COUP-TFII, chicken ovalbumin up-stream transcription factor II.

Second, it has been reported that RA treatment induces lipolysis and depletes lipid storage in mature white adipocytes, which leads to weight loss in diet-induced obese mice [157,158]. All-*trans* RA treatment also decreases white adipose tissue mass in healthy lean mice [159]. The anti-obesogenic effect of all-*trans* RA may be mediated through the activation of PPARβ/δ and RARs, which are important factors in the upregulation of energy dissipation [157,158]. It is also hypothesized that all-*trans* RA promotes the acquisition of brown adipose tissue-like properties in white adipocytes [159]. Indeed, all-*trans* RA induces the expression of uncoupling protein 1 (UCP1), a mediator of adipose thermogenesis (Figure 2), in mouse white adipocytes, possibly via the activation of PPARβ/δ and RARs [160,161,162]. However, all-*trans* RA has no effect, or even inhibits the expression of *UCP1* in human white adipocytes [160]. These conflicting results suggest that other retinoids besides RA may regulate energy metabolism in white adipose tissue. Interestingly, *Raldh1* deficiency has been shown to induce a brown adipose tissue-like transcriptional program in white adipose tissue [163]. Furthermore, retinal can induce the expression of *Ucp1* mRNA and UCP1 protein in white adipose tissue by activating RAR [163]. These data show that both retinal and RALDH1 are regulators of adaptive thermogenesis in white adipose tissue.

Third, RA distinctly influences different stages of adipocyte differentiation. In several mouse preadipocyte cell lines and 3T3-L1 cells, RA can block preadipocyte differentiation through inhibiting the induction of PPARγ and CCAAT-enhancer-binding protein α (C/EBPα) [164]. It has been shown that cellular retinoic-acid binding protein II (CRABP II) mediates the effect of RA [165]. Interestingly, the inhibitory effect of RA on differentiation cannot be observed in the late stage of adipocyte differentiation due to reduced RAR expression levels [166]. Additionally, low doses of all-*trans* RA potentiated the differentiation of Ob17 mouse pre-adipocytes [167].

### 7.2. VA and Metabolism in Brown Adipose Tissue

Brown adipose tissue is engaged in thermogenesis in mammals [168]. It is derived from a cell lineage with a myogenic gene expression signature, which differs from the origin of white adipose tissue [169]. A zinc finger protein, PRD1-BF-1-RIZ1 homologous domain containing protein 16 (PRDM16), induces the expression of a variety of marker genes, which determines brown adipocyte identity and promotes the differentiation of brown preadipocytes into brown adipocytes [170,171]. On the other hand, UCP1 expression levels can be induced in cultured white adipocytes, while the expression of white adipocyte marker genes are retained [172]. Furthermore, beige adipocytes, which are a distinct type of adipocytes with characteristics between white and brown adipocytes, are identified in mouse and human white adipose tissue [173,174,175]. In the human neck region, the adipocytes are aligned in a gradient fashion with the classical brown adipocytes at the inside, beige/brite adipocytes in the middle, and white adipocytes on the outside, which corresponds to the production of heat and usage of fuels [176].

Feeding a VA deficient diet to mice reduced the expression of UCP genes in their brown adipose tissue [177]. In contrast, retinol supplementation induced the expression of *Ucp1* mRNA in rat brown adipocytes [66]. Additionally, in both cultured brown adipocytes and rodent brown adipose tissue, RA treatment stimulated thermogenesis by upregulating the expression of *Ucp1* [177,178,179]. This regulation is mediated through the RARE in the promoter of *Ucp1* gene [180]. It has been shown that all-*trans* RA treatment also reduces RARα and RXRα protein levels in mouse brown adipose tissue [181]. Collectively, these data provide an interesting mechanism by which VA and its metabolites regulate brown adipose tissue thermogenesis.

## 8. VA and Metabolism in the Skeletal Muscle

The skeletal muscle is the largest organ in the body, which plays a critical role in the regulation of energy metabolism [182]. However, very limited research has been performed to investigate the effects of VA on carbohydrate and protein metabolism in the skeletal muscle. In avian species, VA deficiency depletes the glycogen content in the pectoralis major muscle [183]. On the other hand, RA treatment of mouse myoblast C2C12 cells leads to increased glucose uptake, possibly through the activation of AMP-activated protein kinase [184]. In addition, VA deficient rats have lowered protein synthesis and increased proteolysis rates in the skeletal muscle [111,185]. This change in protein metabolism partly contributes to the body mass loss in VA deficient animals. Interestingly, acute VA toxicity accelerates myofibrillar protein breakdown without affecting the rate of protein synthesis, which may also lead to muscle wasting [186]. These data, though sparing, collectively demonstrate the involvement of VA in the regulation of glucose and protein metabolism in the skeletal muscle.

In terms of lipid metabolism, VA deficient rats do not show significant changes in the FA oxidation capacity in the skeletal muscle [123]. In contrast, RA treatment of mouse skeletal muscle dose-dependently increases the transcripts of genes that are involved in FA oxidation and thermogenesis [187]. Indeed, RA treatment and retinyl palmitate supplementation induce the expression of UCP3 in the mouse skeletal muscle [68,157]. Despite the different results observed between rats and mice, these data suggest that VA may increase the energy dissipation in the skeletal muscle.

## 9. Conclusions

VA is a crucial regulator of carbohydrate, protein, and lipid metabolism in all of the major metabolically active organs. The manifold of VA effects may be attributed to the roles of RA (or other VA metabolites) in regulating the expression of critical genes in different metabolic pathways. A lot of effort and progress has been made in understanding the actions of VA (RA) in different organs. We think that further investigations are warranted in the following areas: (1) the molecular mechanisms by which VA regulates the transcription of critical metabolic genes in different organs and tissues, such as *Gck*, *Pck1*, and *Srebp-1c*; (2) the interplay between VA and other hormones in the regulation of metabolic homeostasis; (3) the roles of VA in the regulation of macronutrient metabolism in various disease states, especially obesity, type 2 diabetes, and other metabolic diseases. The findings in the abovementioned areas will not only help understand the effects of micronutrients on energy metabolism, but also help to develop new pharmaceutical strategies to combat metabolic diseases.
